# Quinolinic Acid Responses during Interferon-α-Induced Depressive Symptomatology in Patients with Chronic Hepatitis C Infection - A Novel Aspect for Depression and Inflammatory Hypothesis

**DOI:** 10.1371/journal.pone.0137022

**Published:** 2015-09-14

**Authors:** Andreas Baranyi, Andreas Meinitzer, Robert J. Breitenecker, Omid Amouzadeh-Ghadikolai, Rudolf Stauber, Hans-Bernd Rothenhäusler

**Affiliations:** 1 Department of Psychiatry, Medical University of Graz, Graz, Austria; 2 Clinical Institute of Medical and Chemical Laboratory Diagnostics, Medical University of Graz, Graz, Austria; 3 Alpen-Adria Universität Klagenfurt, Department of Innovation Management and Entrepreneurship, Klagenfurt, Austria; 4 Hospital of the Brothers of St. John of God, Graz, Austria; 5 Division of Gastroenterology and Hepatology, Department of Internal Medicine, Medical University of Graz, Graz, Austria; Macquarie University, AUSTRALIA

## Abstract

**Background:**

The aim of this exploratory study is to gain for the first time a more comprehensive picture of the impact of changes of quinolinic acid concentrations on depressive symptomatology during and after IFN-α therapy.

**Methods:**

The quinolinic acid concentrations of 35 HCV patients are examined in a prospective survey over the entire period of IFN-α treatment as well as three months later at six different times (baseline, one, three, six and nine months after the beginning of IFN-α treatment, and after the end of treatment).

**Results:**

During IFN-α treatment Hamilton Depression Rating Scale scores rise significantly. At the same time there is greater activity of indoleamine 2,3-dioxygenase, with a resulting increase in plasma kynurenine concentrations. Compared to baseline values quinolinic acid concentrations increase significantly during therapy, reflecting an increased neurotoxic challenge. In addition, patients with higher scores in the Hamilton Depression Rating Scale at six and nine months after starting therapy show significantly higher levels of quinolinic acid concentration.

**Conclusions:**

The increase of quinolinic acid during IFN-α therapy might contribute to depressive symptomatology through the neurotoxic challenge caused by quinolinic acid. Subsequently, our exploratory study results support the inflammatory hypothesis of depression. The awareness of relevant risk factors of IFN-α treatment-induced depression is essential to develop preventative treatment strategies.

## Introduction

According to a report by WHO, 3% of the world’s population is estimated to be infected with the hepatitis C virus (HCV), an RNA-virus. Particularly in Eastern European and non-industrialized countries a definite increase of patients with chronic hepatitis has been noted over the past few years. Drug addicts injecting intravenously and hemophiliac patients are particularly at risk of becoming infected with the hepatitis C virus [1–6]. 15% to 45% of the individuals who had contact with HCV are suspected to present spontaneous elimination of the virus. However, up to 55%-85% develop the chronic progressive form of the disease. Possible complications of a chronic hepatitis C infection are the progression to liver cirrhosis and the development of hepatocellular carcinoma [1,2].

Treatment of chronic forms of hepatitis C is based on a standard combination therapy of pegylated interferon-α (IFN-α) and ribavirin. Pegylated IFN-α is a pro-inflammatory cytokine and has strong anti-viral and anti-proliferation characteristics [7]. Ribavirin, an oral nucleoside analog, has broad activity against viral pathogens [8]. The treatment goal of chronic hepatitis C therapy is permanent viral eradication indicated by a sustained virological response. The effectiveness of the antiviral therapy has greatly improved in the last years, and the overall rate of sustained virological response is clearly above 50%. However, while treatment with interferon has shown to be successful, it is also often linked with obvious side effects. Often it is not so much the possible somatic side effects such as thyroid dysfunction, headache and anemia but frequent psychic side effects that impair the health-related quality of life of those affected. Many patients develop a IFN-α-induced depressive symptomatology that usually appears within three months after the start of IFN-α treatment [5,9–13].

Minor depressive episodes with increased introspection, low self-esteem, tearfulness, and loss of libido have been observed through detailed examination in 30% to 60% of all patients treated with IFN-α. 20 to 30% of patients treated with IFN-α even develop the full symptomatology of a major depression, with symptoms such as sadness, loss of energy, interest, and joy, anxiety, insomnia, lack of appetite, impaired concentration, suicidal ideation or suicide attempts. Generally patients affected by depressive symptomatology remit after IFN-α treatment has ended [1,5,14–16]. Adherence to the current standard combination is essential to achieve sustained virological response. The psychic side effects of IFN-α therapy in particular are very stressful and not infrequently lead to termination of the therapy [10, 17].

### Biological pathways for an IFN- α-induced depressive symptomatology

1. IFN-α increases the activity of indoleamine 2,3-dioxygenase (IDO), an enzyme that is responsible for the breakdown of tryptophan to kynurenine. This results in an increase of kynurenine and in reduced concentration of tryptophan that is available for serotonin synthesis [2, 3, 5, 18–20].
2. Kynurenine crosses the blood-brain barrier and is broken down into the neurotoxic metabolites 3-hydroxykynurenines and quinolinic acid. Quinolinic acid has agonistic properties on the N-methyl-D-aspartate (NMDA) receptor. The NMDA receptor is a glutamate receptor, and overstimulation leads to neuronal damage. Animal experiments showed that the intrahippocampal injection of quinolinic acid is linked to a substantial loss of hippocampal neurons. This appears to be particularly important, as major depressive disorder might also be associated with hippocampal volume loss [21–23]. Kynurenic acid is synthesized by the enzyme kynurenine aminotransferase. It is a metabolite of kynurenine and an antagonist of the glutamate recognition site of the NMDA receptor. Thus kynurenic acid is neuroprotective and could prevent NMDA overstimulation. IDO increases the activity of the enzyme kynureninase and hinders the activity of kynurenine aminotransferase. IDO activity increases in the case of INF-α therapy. As a result the breakdown of kynurenine is steered in the direction of neurotoxic quinolinic acid, and the concentration of neuroprotective kynurenic acid decreases [21, 23, 24].

### Aims of the study

This exploratory study is part of our research project on interferon-α-induced depressive symptomatology. The aim of this research project is to gain a more comprehensive picture of the kynurenine pathway in HCV patients before, during and after IFN-α therapy. A biopsychosocial model of interferon-α-induced depression was already developed in the first published study [5].

Our new study is the first prospective study to assess whether increased quinolinic acid concentrations during IFN-α therapy are linked to depressive symptomatology. Especially for this study our research group developed a new and validated simple liquid chromatography-tandem mass spectrometric method for the determination of quinolinic acid, which is described in detail elsewhere [25]. The quinolinic acid concentrations are measured at six different times before, during and after the end of IFN-α treatment.

## Materials and Methods

### Participants

The study design is partly described elsewhere [5] but is more extensively detailed here. All patients with chronic hepatitis C infection who had been selected for IFN-α therapy during the study period (February 2009–January 2012) by the hepatologist (R.S.) were asked to participate in this research project.

For this second study a subgroup of patients free of illegal drugs was set up, as the consumption of illegal drugs might influence quinolinic acids concentrations. Thus, the reasons for exclusion from enrolment were expanded in this study and included (1) other chronic liver disease, (2) significant co-morbid conditions (i.e. cardiovascular disease, cancer), (3) acute depression, (4) a diagnosis of neurological disease, and (5) current illegal drug or substance abuse, or participation in a federal drug substitution program. 35 patients were enrolled in this prospective study. All patients were treated in the outpatient clinic of the Division of Gastroenterology and Hepatology, Department of Internal Medicine, Medical University of Graz, Austria and received pegylated IFN-α treatment (peginterferon-α 2a or peginterferon-α 2b) in combination with ribavirin. Both kinds of interferons have nearly the same efficacy, and bring about the same activation of the kynurenine pathway [14]. In this study 29 (82.9%) patients received IFN-α 2a, and 6 (17.1%) patients were treated with IFN-α 2b. Psychiatric and biological assessments were carried out at six different times: before, during (at one, three, six and nine months), and after the end of IFN-α treatment.

The study was approved by the Institutional Review Board of the University of Medicine of Graz, Austria. Data protection met the standards set by Austrian law. All participants in this study gave signed informed consent.

### Biological Assessments

Blood was sampled from the fasting subjects between 0900 and 1000 at baseline, one, three, six and nine months after the beginning of the IFN-α therapy, and three months after the end of treatment for the assay of tryptophan, kynurenine and quinolinic acid.

For the research project tryptophan and kynurenine were measured in plasma samples by high-performance liquid chromatography (HPLC) with a simultaneous ultraviolet and fluorometric detection system [5, 26, 27]. In brief, 100 μL plasma sample was deproteinized by adding 100 μL of 5% (v/v) perchloric acid. After vortexing and 5 min centrifugation at 11,000 g, 20 μl of the clear supernatant was injected in the chromatographic system. Separations were achieved on a Chromolith RP18e column (100 x 4.6 mm, 5 μm, Merck Darmstadt, Germany) at 30°C by isocratic elution with a mobile phase (pH 4.9) consisted of 50 mmol/l ammonium acetate, 250 mol/L zinc acetate and 3% (v/v) acetonitrile, at a flow-rate of 0.8 ml/min. Kynurenine and tryptophan were detected on a La Chrom UV-Detector Merck HITACHI L-7400; at 235 nm. Capture and processing of the chromatograms are performed using Merck HITACHI LaChrom-D-7000 HPLC-System-Manager-Software (VWR International GmbH/Scientific Instruments, Darmstadt). The concentrations were determined as the peak measurement against external standards. The method is well established in the Clinical Institute of Medical and Chemical Laboratory Diagnostics, Medical University of Graz, Austria, and validated according to international guidelines. All reagents were p.A. grade from Merck (Darmstadt, Germany). Coefficients of variation (CVs) at different concentrations within the same day were in the range of 1.7%-4.3% for kynurenine and 0.7%-2.9% for tryptophan. The CVs between different days were 2.0%-5.4%, 6.3%-9.3% and 8.4%-11.6% respectively.

We developed a new and validated liquid chromatography-tandem mass spectrometric method for the determination of quinolinic acid, which is described in detail elsewhere [25]. Quinolinic acid was measured in frozen EDTA plasma [25, 28]. Within-day coefficients of variation (CVs) and between-day CVs were all below 10%.

### Psychiatric Assessments

As already shown in the first study [5], all consenting patients were interviewed before, during (at one, three, six and nine months after the beginning of the IFN-α treatment) and three months after the end of IFN-α therapy by experienced consultant-liaison psychiatrists (A.B., H.-B. R.). At every date of examination all participating patients were examined for the presence of depressive symptoms by using the psychometric observer-rated scale Hamilton Depression Rating Scale (HAMD-17; [29]).

1. **Sociodemographic, HCV Infection and Treatment Characteristics Questionnaire.** Collected sociodemographic variables included age, gender, years of education and/or vocational training, and marital status at the time of psychiatric assessment. Marital status was categorized as single, married, or widowed.
  Clinical and treatment characteristics were: subtype of chronic HCV infection, presumed means of transmission, type of IFN-α, dosage of IFN-α, length of time of IFN-α treatment, concomitant pharmaceutical treatment (e.g. ribavirin), and success of treatment.
2. **Hamilton Depression Rating Scale (HAMD-17).** The Hamilton Rating Scale for Depression (HAMD-17, Hamilton, 1967) consists of 17 items and is one of the most used instruments for the diagnosis of depressive symptomatology. A score of 18 points or more indicates severe depression, the range of 14–17 points indicates moderate depression, and the range of 10–13 points indicates mild depressive symptomatology [29].

### Statistical Analyses

Descriptive statistics were produced based on demographic, treatment-related, biochemical and psychometric data and are presented as mean and standard deviation (SD). Repeated measure design ANOVA (RM-ANOVA) was used to check the effects of IFN-α on Hamilton Depression Rating Scale scores, indoleamine 2,3-dioxygenase (IDO) activity, kynurenine and quinolinic acid concentrations during and after treatment. Furthermore, we performed a bivariate Pearson product-moment correlation between Hamilton Depression Rating Scale scores and quinolinic acid concentrations. All statistic tests were two-tailed, with significance set at p<.05. Due to the exploratory character of this study the results were not α adjusted for multiplicity. All statistical analyses were performed with the R Project for Statistical Computing (R Development Core Team, 2011) and SPSS 20.0 for Windows (IBM-SPSS Statistics).

## Results

### Sociodemographic and Treatment Characteristics, Presumed Means of Transmission

All participating 35 HCV patients (14 [40%] women, 21 [60%] men) were Caucasian, and the mean age was 46.8 years (SD = 12.7).

In 17 (48.6%) patients the presumed means of transmission was unknown. 15 (42.9%) reported a history of transfusion or medical interventions as a source of HCV infection, and in 3 (8.6%) patients, previous intravenous drug abuse was the presumed means of transmission.

Table 1 summarizes the sociodemographic, clinical and treatment characteristics of the whole sample, including the type of hepatitis, the prescribed medication, the dosage of IFN-α, the duration of IFN-α therapy, and the frequency of treatment success for the examined drug-free subgroup.

**Table 1:** Sociodemographic and treatment characteristics.

Category	All patients (n = 35)	p
***Gender***		p = 0.240 ^a^
** Male**	21 (60%)	
** Female**	14 (40%)	
***Age***		
** Mean (years)**	46.8	
** SD**	12.7	
***Marital status***		p = 0.060 ^a^
** Single**	12 (34.3%)	
** Married**	13 (37.1%)	
** Widowed**	3 (8.6%)	
** Divorced**	7 (2.9%)	
***Employment status***		p = 0.13 ^a^
** Paid work**	22 (62.9%)	
** No paid work (homeworker, unemployed, retired)**	13 (37.1%)	
***Maximum education level***		p<0.001 ^a^
** Elementary school**	4 (11.4%)	
** Apprenticeship**	18 (51.4%)	
** College**	6 (17.1%)	
** Technical/Trade school**	3 (8.6%)	
** University**	4 (11.4%)	
***Living arrangements***		p<0.001 ^a^
** Alone**	15 (42.9%)	
** With others (family, partner or friends)**	20 (57.1%)	
***History of depressive disorder***		p = 0.028 ^a^
** Yes**	11 (31.4%)	
** No**	24 (68.6%)	
***History of* drug abuse**		p<0.001 ^b^
** Yes**	4 (11.4%)	
** No**	31 (88.6%)	
***Way of transmission***		p<0.01 ^a^
** Unknown**	17 (48.6%)	
** History of transfusion**	15 (42.9%)	
** Intravenous drug abuse**	3 (8.6%)	
***Type of Hepatitis C infection***		p<0.001 ^a^
** HCV Type 1**	7 (20%)	
** HCV Type 1a**	7 (20%)	
** HCV Type 1b**	15 (42.9%)	
** HCV Type 2a**	1 (2.9%)	
** HCV Type 3**	4 (11.4%)	
** HCV Type 4**	1 (2.9%)	
***Exposure to any prior IFN-α treatment***		p<0.001 ^a^
***0***	19 (54.3%)	
***1***	11 (31.4%)	
***2***	4 (11.4%)	
***3***	1 (2.9%)	
***Type of interferon***		p<0.001^b^
** Peg INF-α-2a**	29 (82.9%)	
** Peg INF-α-2b**	6 17.1%)	
***Dosage***		
** Peg INF-α-2a**	180.0 μg	
** Range**	45	
** Peg INF-α-2b**	100.0 μg	
** Range**	0	
***Duration of treatment***		
** Mean (weeks)**	36.63	
** SD**	19.02	
***Success of treatment***		p<0.223 ^a^
** Yes**	13 (39.4%)	
** No**	20 (60.6%)	

SD = Standard deviation

^a^ χ^2^ test

^b^ Fisher's exact test

### Depressive Symptomatology

In our first study [5] we were able to show that patients receiving IFN-α treatment suffer more often from depressive symptoms. This also applies to the drug-free patients of this examined subgroup. Thus while during IFN-α treatment the scores in the Hamilton Depression-Rating Scale increased significantly in the whole group of drug-free patients and after the end of IFN-α therapy, HAMD-17 total scores in all patients returned to values similar to the baseline measurements before IFN-α treatment (HAMD-17: RM-ANOVA[time]: F[5,134] = 10.45, p<0.001). In patients with IFN-α therapy depressed mood (HAMD-17) significantly increased during IFN-α treatment and returned to values similar to the baseline measurements after the end of treatment (RM-ANOVA [time]: F[5,134] = 7.921, p<0.001). Depressive symptomatology displayed during therapy but not before lasted from several weeks up to several months.

Table 2 presents the Hamilton Depression Rating Scale (HAMD-17) scores before, during and three months after IFN-α treatment for the examined drug-free subgroup.

**Table 2:** Presents the Hamilton Depression Rating Scale (HAMD-17) scores before, during and three months after IFN-α treatment for the examined drug-free subgroup. Means and standard deviations of Hamilton Depression Rating Scale (HAMD-17) scores, indoleamine 2,3-dioxygenase (IDO) activity, kynurenine and quinolinic acid concentrations before, during and after IFN-α therapy.

	Baseline		1 month		3 months		6 months		9 months		3 months after IFN-α therapy		p ^1^
	Mean	SD	Mean	SD	Mean	SD	Mean	SD	Mean	SD	Mean	SD	
**HAMD-17 Total Score**	2.86	3.06	7.46	5.77	7.48	6.72	6.81	6.60	6.59	6.32	2.96	3.65	RM-ANOVA [time]: F[5,134] = 10.45, **p<0.001**
HAMD-17 Depressed Mood	0.09	0.28	0.80	1.08	0.97	1.17	0.88	1.18	0.76	1.09	0.22	0.51	(RM-ANOVA [time]: F[5,134] = 7.921, p<0.001)
**IDO-activity** (Kynurenine/Tryptophan x 1000)	46.50	13.02	51.74	10.78	59.32	14.78	65.0	18.35	72.15	20.66	45.46	12.04	RM-ANOVA [time]: F[5,120] = 18.99, **p<0.001**
**Kynurenine (μmol/L)**	2.49	0.71	2.59	0.56	2.72	0.73	2.88	0.65	3.16	0.46	2.55	0.68	RM-ANOVA [time]: F[5,126] = 5.34, **p<0.001**
**Quinolinic acid (nmol/L)**	524.97	151.82	543.94	133.49	544.12	161.30	629.59	286.03	674.05	311.02	506.61	177.98	RM-ANOVA [time]: F[5,128] = 3.48, **p = 0.005**

^1^ RM-Anova: [before, during IFN-α therapy]

### Indoleamine 2,3-dioxygenase (IDO) Activity

A significant increase in the kynurenine/tryptophan (KYN/TRYP) ratio reflecting IDO activity could also be observed in the examined subgroup of drug-free patients. Thus the kynurenine/tryptophan (KYN/TRYP) ratio increased significantly in all patients compared to baseline at all time points during IFN-α therapy, suggesting an increased catabolism of tryptophan to kynurenine (KYN/TRYP ratio: RM-ANOVA[time]: F[5,120] = 18.99, p<0.001). In addition, kynurenine concentrations increased significantly in all patients during IFN-α treatment compared to baseline (kynurenine: RM-ANOVA[time]: F[5,126] = 5.343, p<0.001).

Table 2 summarizes the indoleamine 2,3-dioxygenase (IDO) activity and kynurenine concentrations before, during and three months after IFN-α treatment for the examined drug-free subgroup.

### Quinolinic Acid

Compared to baseline values, quinolinic acid concentrations increased significantly during IFN-α therapy, with peak concentrations six and nine months after the start of treatment. Three months after the end of IFN-α therapy quinolinic acid concentrations returned to values similar to baseline concentrations (quinolinic acid: RM-ANOVA[time]: F[5,128] = 3.48, p = 0.005). Furthermore, patients with higher scores in the HAMD-17 Depression Scale at six and nine months after starting therapy also showed significantly higher levels of quinolinic acid concentrations.

Table 2 shows the quinolinic acid concentrations before, during and three months after IFN-α treatment.

Table 3 presents the bivariate Pearson product-moment correlation coefficients between quinolinic acid concentrations and Hamilton Depression Rating Scale (HAMD-17) scores during and after IFN-α therapy.

**Table 3:** Bivariate Pearson product-moment correlation coefficients between quinolinic acid concentrations and Hamilton Depression Rating Scale (HAMD-17) scores before, during and after IFN-α therapy.

	HAMD-17—baseline		HAMD-17—1 month		HAMD-17—3 months		HAMD-17—6 months		HAMD-17—9 months		HAMD-17—3 months after therapy	
	*r*	*p*	*r*	*p*	*r*	*p*	*r*	*p*	*r*	*p*	*r*	*p*
**Quinolinic acid—baseline**	0.144	*0*.*426*										
**Quinolinic acid—1 month**			-0.094	0.590								
**Quinolinic acid—3 months**					-0.007	0.968						
**Quinolinic acid—6 months**							**0.435**	**0.034**				
**Quinolinic acid—9 months**									**0.566**	**0.028**		
**Quinolinic acid—3 months after therapy**											-0.118	0.565

## Discussion

Depressive symptomatology is a frequent side effect of IFN-α therapy in patients with chronic hepatitis C infection. In the drug-free subgroup examined for this study the depression scores measured according to HAMD-17 during IFN-α therapy also increased significantly, and a return to baseline depression scores after the end of IFN-α therapy was observed. Previous studies have reported similar increases in depressive symptomatology during IFN-α therapy [1, 14–16]. IFN-α increases the activity of indoleamine 2,3 dioxygenase (IDO) [5]. As expected, the kynurenine/tryptophan ratio reflecting IFN-α-induced IDO activity increased significantly in the drug-free subgroup, suggesting an increased catabolism of tryptophan to kynurenine. This results in an increase of plasma-kynurenine and a decrease of plasma-tryptophan during IFN-α therapy. The actual quinolinic acid concentrations over the entire duration on the therapy and three months after were determined for the first time in this subsequent study, and the results of our study show an increased neurotoxic challenge in drug-free patients during IFN-α therapy. Thus in our study the quinolinic acid concentrations increased significantly during IFN-α therapy and returned to baseline concentrations after end of therapy. The peak values of quinolinic acid were reached six and nine months after the start of the IFN-α therapy. In addition, there was a significant positive correlation between quinolinic acid and the depression scores on the Hamilton Depression Rating Scale at both these times, indicating a higher risk of depressive symptomatology through the neurotoxic challenge caused by the increase of the quinolinic acid. These results are strong evidence, that higher concentrations of quinolinic acid might be essential for the evolution of quinolinic acid induced depressive symptoms. Wichers et al. [23] examined 16 patients undergoing interferon treatment. The total Montgomery-Åsberg Depression Rating Scale score assessed in this study was significantly associated over time with the kynurenine/kynurenic acid ratio. This ratio had been used earlier as an indirect estimation of increased neurotoxic challenge due to the synthesis of neurotoxic kynurenine metabolites. This increased neurotoxic challenge under IFN-α therapy was also confirmed by our research group [5]. Raison et al. [18] could prove in a cross-sectional assessment 12 weeks after the start of IFN-α therapy that patients with chronic HCV-infection had a higher concentration of quinolinic acid in their cerebrospinal fluid in comparison with untreated controls. Even in patients with acute depression not induced by IFN-α, many studies show a specific increase of monocyte-derived cytokines (Il-1, Il-6, TNF-α) and abnormalities of lymphocytes and natural killer cells in the peripheral blood. These neuroinflammatory responses also show up in specific regions of the brain through increased density of microglial cells, which represent the mononuclear phagocyte system of the brain [30,31]. Corresponding with these findings Steiner et al. [30] reported an upregulated production of quinolinic acid by microglia in specific brain regions like the subgenual anterior cingulate cortex and the anterior midcingulate cortex in postmortem brains of acutely depressed patients who had committed suicide. These results might support the immuno- and neurodegeneration hypotheses of depression [30,31]. Quinolinic acid can cause acute or chronic neuronal dysfunction through nine already known mechanisms. (a.) Quinolinic acid is an agonist of the N-methyl-D-aspartate (NMDA) receptor and activates the NMDA receptor in pathophysiological concentrations. The consequence is massive calcium entry into neurons. Neurons in the hippocampus, striatum and neocortex are especially sensitive to quinolinic acid, and these brain areas contain a particularly high number of NMDA receptors [32–35]. (b.) Quinolinic acid could cause disruption of the integrity of the blood-brain barrier [30]. (c.) Quinolinic acid can cause greater glutamate release by neurons and inhibits its reuptake by astrocytes. Excessive microenvironment glutamate concentrations cause neurotoxicity [36, 37]. (d.) A complex of quinolinic acid and iron transfers an electron to oxygen. Thus, reactive oxygen types are formed which mediate lipid peroxidation [38–43]. (e.) Quinolinic acid can potentiate the toxicity of other excitotoxins (e.g. glutamate, glycin and NMDA). The result is progressive mitochondrial dysfunction [44]. (f.) Quinolinic acid may impair autophagy [45]. (g.) Quinolinic acid may lead to cytoskeleton destabilization, causing intermediate filament hyperphosphorylation [46–48]. h.) Quinolinic acid is involved in the dysregulation of astroglial function and gliotoxicity [49, 50]. In addition Guillemin et al. [45] showed that quinolinic acid selectively induces apoptosis of human astrocytes. Astrocytes are known to produce neuroprotective kynurenic acid. As a consequence the apoptosis of astrocytes might lead to lower neuroprotective action against neurotoxic quinolinic acid [51, 52]. (i.) Finally, the neuronal nitric oxide synthase and the inducible nitric oxide synthase may be induced by quinolinic acid. As a consequence, free radical production and oxidative stress are the result of quinolinic acid-induced NOS activity in astrocytes [45, 51].

### The Biopsychosocial Factor

There is growing evidence that the etiology of IFN-α induced depression is multifactorial in nature. Quinolinic acid might contribute as one biological key factor to depressive symptoms in patients during IFN-α therapy. In the simultaneously presence of additionally known biopsychosocial factors (e.g. tryptophan availability, gender, preexisting psychiatric vulnerability, means of transmission, low financial security, impaired sexual satisfaction, small circle of friends, impaired physical role, body pain, low general health and vitality, reduced social functioning, impaired mental health and impaired emotional role) depressive symptoms may aggravate to clinical significance or even a depressive episode may become manifest [5]. Thus the impact of quinolinic acid should always be seen in a biopsychosocial context.

### Limitations

The indolamine 2,3- dioxygenase (IDO) activity in the human blood can only be described in an indirect way regarding the trytpophan to kynurenin ratio. The expression of IDO is differently inducible in various cell types, including fibroblasts, monocytes, macrophages, and dendritic cells by the pro-inflammatory cytokine interferon-γ. For an exact determination of IDO activity it would be necessary to individually isolate IDO from these various cell types. In the scientific literature, the trytpophan to kynurenin ratio is considered to be a suitable biomarker for IDO activity [3, 23, 53].

During therapy there is an increase of quinolinic acid and of HAMD-17 depression values. This concurrent increase therefore allows for an assertion of an associative correlation. However, theoretical considerations allow for the hypothesis that quinolinic acid increases the risk of depressive symptomatology through its neurotoxic potency. This possible causal link must be clearly established in further confirmatory studies. More studies are required to determine any set patterns. Furthermore, knowledge about the complex interactions of kynurenine pathway, IFN-α treatment and depressive symptomatology is still limited and requires ongoing research.

### Conclusions

The increase of quinolinic acid during IFN-α therapy might contribute to depressive symptomatology through the neurotoxic challenge caused by quinolinic acid. Subsequently, our exploratory study results support the inflammatory hypothesis of depression. The awareness of relevant risk factors of IFN-α treatment-induced depression is essential to developing preventative treatment strategies.

## Supporting Information

S1 Fig The Tryptophan Metabolism. (PDF)

S1 Dataset (XLSX)

## Abbreviations

3-HK: 3-hydroxykynurenine
HAMD-17: Hamilton Rating Scale for Depression
HCV: hepatitis C virus
IDO: indoleamine 2,3-dioxygenase
IFN-α: interferon-α
NMDA: N-methyl-D-aspartate
RM-ANOVA: repeated measure design analysis of variance
SD: standard deviation
