# Supplementary figures and images for: Quinolinic Acid Responses during Interferon-α-Induced Depressive Symptomatology in Patients with Chronic Hepatitis C Infection - A Novel Aspect for Depression and Inflammatory Hypothesis
Source: PLoS One. 2015 Sep 14;10(9):e0137022. doi: 10.1371/journal.pone.0137022 (PMC4569409; doi:10.1371/journal.pone.0137022)

# S1 Figure: The Tryptophan Metabolism

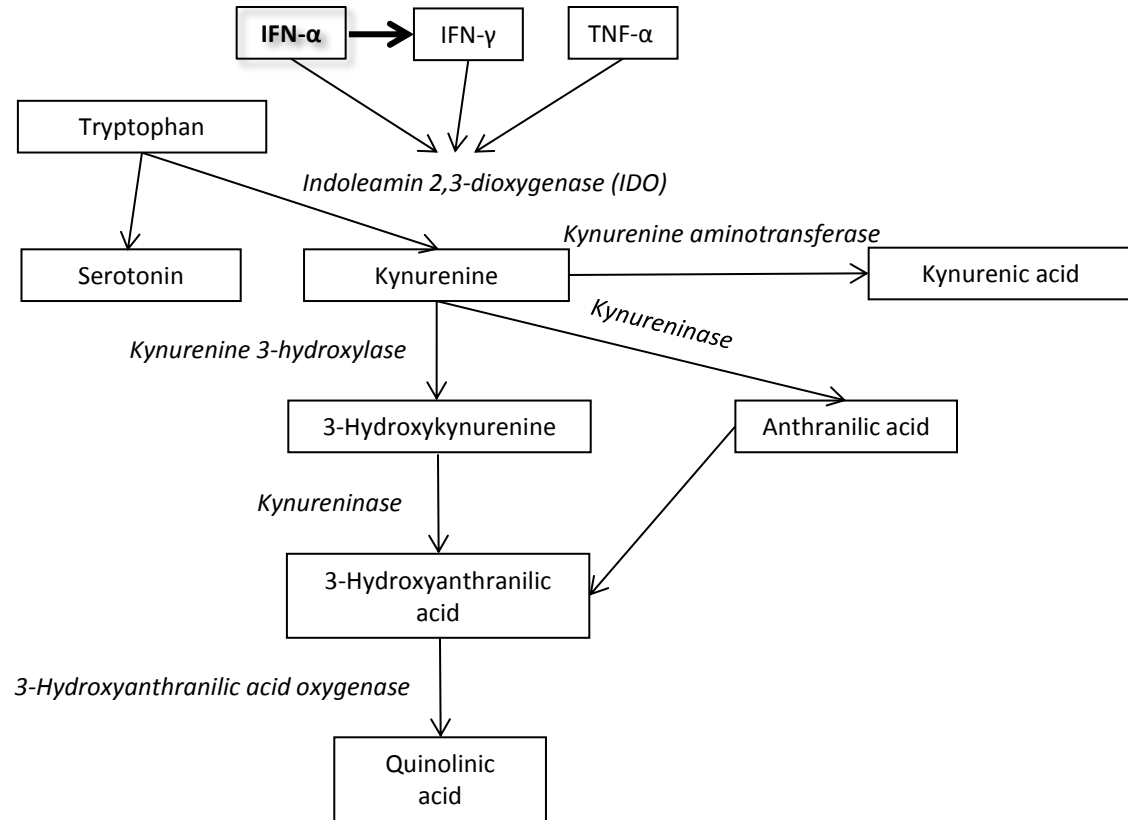

Supplement: S1 Fig — (PDF) [file pone.0137022.s001.pdf]
